# A linear model for event-related respiration responses

**DOI:** 10.1016/j.jneumeth.2016.06.001

**Published:** 2016-09-01

**Authors:** Dominik R. Bach, Samuel Gerster, Athina Tzovara, Giuseppe Castegnetti

**Affiliations:** a Department of Psychiatry, Psychotherapy, and Psychosomatics, University of Zurich, Switzerland; b Neuroscience Center Zurich, University of Zurich, Switzerland; c Wellcome Trust Centre for Neuroimaging, University College London, United Kingdom

**Keywords:** Respiration, Gas exchange, Respiration rate, Tidal volume, Minute volume, Evoked responses, Psychophysiology, Psychophysiological Model

## Abstract

**Background:**

Cognitive processes influence respiratory physiology. This may allow inferring cognitive states from measured respiration. Here, we take a first step towards this goal and investigate whether event-related respiratory responses can be identified, and whether they are accessible to a model-based approach.

**New method:**

We regard respiratory responses as the output of a linear time invariant system that receives brief inputs after psychological events. We derive average responses to visual targets, aversive stimulation, and viewing of arousing pictures, in interpolated respiration period (RP), respiration amplitude (RA), and respiratory flow rate (RFR). We then base a Psychophysiological Model (PsPM) on these averaged event-related responses. The PsPM is inverted to yield estimates of cognitive input into the respiratory system. This method is validated in an independent data set.

**Results:**

All three measures show event-related responses, which are captured as non-zero response amplitudes in the PsPM. Amplitude estimates for RA and RFR distinguish between picture viewing and the other tasks. This pattern is replicated in the validation experiment.

**Comparison with existing methods:**

Existing respiratory measures are based on relatively short time-intervals after an event while the new method is based on the entire duration of respiratory responses.

**Conclusion:**

Our findings suggest that interpolated respiratory measures show replicable event-related response patterns. PsPM inversion is a suitable approach to analysing these patterns, with a potential to infer cognitive processes from respiration.

## Highlights

• We develop a novel method for analysing event-related respiratory responses.
• This method is based on a Psychophysiological Model (PsPM) of interpolated time series.
• We analyse respiration period (RP), amplitude (RA) and flow rate (RFR).
• RA and RFR estimates distinguish different event types, and all three measures distinguish events from non-events.
• The new method could be useful for fMRI experiments using respiration belts.

## 1 Introduction

Brain stem centres regulate respiration via autonomic nervous efferents, and these centres are influenced by higher cognitive processes (Lorig, 2007, Ritz et al., 2010, Wientjes and Grossman, 1998). A rich psychobiological literature has addressed how cognitive states impact respiration patterns (Grassmann et al., 2015, Vlemincx et al., 2014, Wuyts et al., 2011), gas exchange parameters (Grassmann et al., 2015), and airway responses (Ritz et al., 2010, Van Diest et al., 2009), and how this may contribute to pathologies such as in asthma (Ritz et al., 2014) or panic disorder (Grassi et al., 2014). In turn, such psychophysiological relationship may allow inferring cognitive states from measured respiration. It is for example known that autonomically controlled skin conductance responses (SCR) (Boucsein, 2012) or heart period responses (HPR) (Bradley et al., 2001) are informative about psychological processes. The analysis of such signals has been formalised in the context of Psychophysiological Modelling (PsPM) (Bach and Friston, 2013). A PsPM is usually a combination of a formal (mathematical) model for the neural activity that links psychological processes to the physiological signal under study (neural model), and another model that specifies the relation between neural activity and physiological signal (peripheral model). The combined model is probabilistically inverted to yield the most likely parameters of the neural activity, given physiological data. These parameters characterise the presumed psychological or cognitive input into the system.

Here, we seek to create a PsPM for the relationship between central input and respiratory responses. Crucially, the aim of this PsPM is not to precisely characterise respiratory physiology, but to characterise cognitive states by inversion of the model. This means that even very simple respiratory measures, which may not allow precise quantification of local physiology such as gas exchange or respiration patterns, can be useful as long as they are informative about a psychological process. In cognitive neuroscience research, magnetic resonance imaging (MRI) scanners are standardly endowed with single chest belts for correcting breathing artefacts in MR images (Glover et al., 2000, Hutton et al., 2011). This motivates our present work where we seek to employ this simple measurement system for inferring cognitive states. If successful, this method could thus be harnessed for analysis of a large number of existing datasets, and allow future investigation with existing setups.

In general, there are two methods to develop a PsPM. If the physiological system under study is well-characterised, one may employ such knowledge to create a biophysical model of psychological influences on peripheral physiology, such as the haemodynamic response model in neuroimaging (Friston et al., 2008). More often, however, this is not the case. Alternatively, one may attempt a phenomenological characterisation of the system's input–output relationship. For example, by assuming linearity and time invariance, one can use brief inputs to derive the system's impulse response function. This approach has been fruitful in the context of SCR and HPR (Bach et al., 2009, Bach et al., 2010b, Paulus et al., 2016). Therefore, respiratory responses to brief stimuli would be of primary interest for a respiratory PsPM. As yet, most studies in the field of respiratory psychobiology – including all of the aforementioned work – have addressed respiratory responses to states or stimuli on the timescale of at least 10–20 s up to minutes, or responses to (anticipated) respiratory stimuli (Pappens et al., 2015). However, a few strands of research indicate that an organism's interaction with non-respiratory events on a much shorter time scale also impacts on respiration.

First, the existence of a respiratory orienting response has been proposed by Barry (1977b) who related its magnitude to stimulus novelty (Barry, 1977a). This relationship was confirmed by quantifying respiratory breaks as the duration of the respiration cycle during which a stimulus was presented, measured from start of inspiration (Barry, 1982). This novelty response has later also been termed “surprise” response (Boiten et al., 1994) but has not systematically been investigated after the original proposal. Secondly, intense, unexpected aversive stimulation may elicit “a short-latency [inspiratory] startle response, followed by a delayed phasic increase in depth and rate of breathing” (Boiten et al., 1994). Finally, respiration line length (RLL), quantified as the path length of the respiration trace over a fixed time interval of usually 15 s after an event, has been suggested to differentiate between crime-relevant and crime-irrelevant items in the concealed information test, one of the few validated tests for detection of deception in the polygraph field (Matsuda and Ogawa, 2011).

Taken together, this suggests that respiratory breaks, and phasic changes in respiratory period and amplitude, might be informative about cognitive processes. The single-belt system employed here allows assessing respiration timing, while for precise quantification of respiratory amplitude, a double-belt system would be required to measure both thoracic and abdominal compartments (Binks et al., 2007). However, if the ratio between thoracic and abdominal contribution is relatively constant within any individual, it may still be possible to approximate respiratory amplitude up to a linear constant from the single-belt system. This is why we ask empirically whether measures of respiration amplitude allow a meaningful inference on psychological state.

We propose a PsPM approach based on continuous data and linear time-invariant systems, as in previous work on skin conductance (Bach et al., 2009, Bach et al., 2010b, Bach and Friston, 2013, Bach et al., 2010c) and heart period responses (Castegnetti et al., 2016, Paulus et al., 2016). We are interested in evoked responses to events in the outside world that are non-synchronised to respiration. This means that time after stimulus onset does not correspond to particular fixed time points in the respiration cycle. To solve the problem of assigning respiratory-cycle based measures to real time, we follow a strategy commonly employed in analysis of heart period responses, namely, linear interpolation (Berntson et al., 2007). We capitalise on an established modelling framework (Bach and Friston, 2013), to build a phenomenological forward model of how cognitive input impacts respiration period. This forward model is combined with a model inversion method. This provides for inference on the amplitude of central input from measured data, and is embodied in a general linear convolution model (GLM). All algorithms are publicly available as part of a Matlab toolbox for Psychophysiological Modelling, PsPM (previously termed SCRalyze, http://pspm.sourceforge.net).

## 2 Method

### 2.1 Participants

We recruited from the adult student population via advertisements 30 participants for experiments 1–2 (23 female, mean age ± standard deviation: 23.4 ± 3.6 years). From this sample, 5 persons did not participate in the electric stimulation task 2, and 4 datasets from task 1 were discarded due to marker malfunction, such that we report 26 datasets for experiment 1 and 25 datasets for experiment 2. Twenty participants took part in experiment 3 (12 female, 22.2 ± 3.6 years), and an independent sample of 20 participants in validation experiment 4 (9 female, 25.3 ± 5.1 years). In experiment 3 we also recorded heart period which was included in a previous methodological investigation (Paulus et al., 2016). All experiments and the form of taking written informed consent were approved by the competent research ethics committee (Kantonale Ethikkomission Zürich, KEK-ZH Nr. 2013-0118 and 2013-0258).

### 2.2 Procedure

#### 2.2.1 General considerations

We were interested in characterising phasic respiratory changes that differentiate different experimental events, which we selected with an eye on previous findings. One dimension supposedly eliciting phasic respiratory changes is stimulus *novelty*, operationalised previously by repeating simple auditory or visual stimuli in a detection task (Barry, 1977a, Barry, 1982). Experiment 1 therefore realised a visual detection task with 10 target repetitions. Another relevant class are *intense, aversive* stimuli (Boiten et al., 1994). This was realised in experiment 2 using unpredictable and discomforting electric stimulation. Finally, *crime-relevance* is a dimension known from applied psychology to elicit phasic respiratory responses (Matsuda and Ogawa, 2011), and is possibly related to emotional arousal. As we were not interested in crime-relevance as such, we investigated emotional arousal by showing pictures with high and low arousal ratings in experiment 3. In all 3 experiments, events were not synchronised with respect to respiration, as we were interested in the applicability to generic experimental circumstances. Finally, since we did not know how long the tail of possible respiratory responses would be, and since other autonomic responses (skin conductance and heart period responses) have tails that extend over more than 30 s, we used long intervals (>40 s) between subsequent events of interest. Experiment 4 aimed at replicating findings from experiments 1–3.

#### 2.2.2 Tasks and stimuli

In Experiment 1 (dataset code RRM2), participants saw a train of 570 grey distractor digits on the screen and were instructed to press a key when they detected one of 10 interspersed red "+" targets. The targets in this task have previously been shown to induce phasic sympathetic arousal, while the other stimuli (i.e. the distractors) did not induce sympathetic responses (Bach et al., 2010b). Each stimulus (targets and distractors) was presented for 200 ms and separated from the next stimulus by an 800 ms inter stimulus interval. The experiment was divided into two blocks, a baseline block and a target block. The order of the two blocks was balanced across participants, and the transition between the two blocks was not signalled. The baseline block consisted of 80 distractors and 1 target at the end, and was not analysed here. In the target block, inter target interval (ITI) was randomly drawn from 40 s, 45 s, or 50 s, and the first target was preceded by 20 distractors.

In experiment 2 (dataset code RRM1), participants were exposed to 10 electric stimulations with an ITI randomly drawn from 40 s, 45 s, or 50 s. Stimulations consisted of a 500 ms train of square pulses with 2 ms period and 10% duty cycle, delivered via a pin-cathode/ring-anode configuration attached to the dominant forearm (Digitimer DS7A). Pulse intensity was individually determined before the experiment to be just below the pain threshold and clearly uncomfortable.

In experiment 3 (dataset code HRM_IAPS), participants watched the 16 least arousing neutral, 16 most arousing aversive, and 16 most arousing pleasant (excluding explicitly nude) pictures from the International Affective Picture System (IAPS; Lang et al., 2005) in randomised order. The selected pictures were the same as in previous studies (Bach, 2014, Bach et al., 2013, Bach et al., 2015). Participants were instructed to press the cursor up or down key on a computer keyboard to indicate whether they liked or disliked the picture. Pictures were presented for 1 s with an ITI randomly drawn from 43 s, 45 s or 47 s. All pictures were presented in one block.

In experiment 4 (dataset code RRM3), participants were presented with 60 stimuli from 4 conditions in randomised order: the 15 most arousing aversive, and 15 most arousing pleasant (excluding explicit nude) pictures from the International Affective Picture System (IAPS; Lang et al., 2005), and white noise sounds of either 65 dB intensity (15 stimuli), or 85 dB intensity (15 stimuli). All stimuli were presented for 1 s with an ISI randomly drawn from 40 s, 45 s or 50 s; the experiment started with a 5 s rest period.

#### 2.2.3 Data recording

A bellows belt was fitted around the rib cage over the lower end of the sternum. The bellows was connected to a pressure transducer (aneroid chest bellows, V94-19, Coulbourn Instruments) and amplified (V72-25B, Coulbourn Instruments). The signal was A/D converted with a sampling frequency of 1000 Hz and recorded (DI-149/Windaq, Dataq).

### 2.3 Data analysis

All data analysis was done in Matlab and using PsPM 3.0.2.

#### 2.3.1 Data preprocessing

Transducer output was filtered offline with an anti-aliasing bidirectional first-order Butterworth low-pass filter and a cut-off frequency of 5 Hz. Data were then downsampled to 10 Hz resolution.

#### 2.3.2 Detection of breathing cycles

**Fig. 1:**
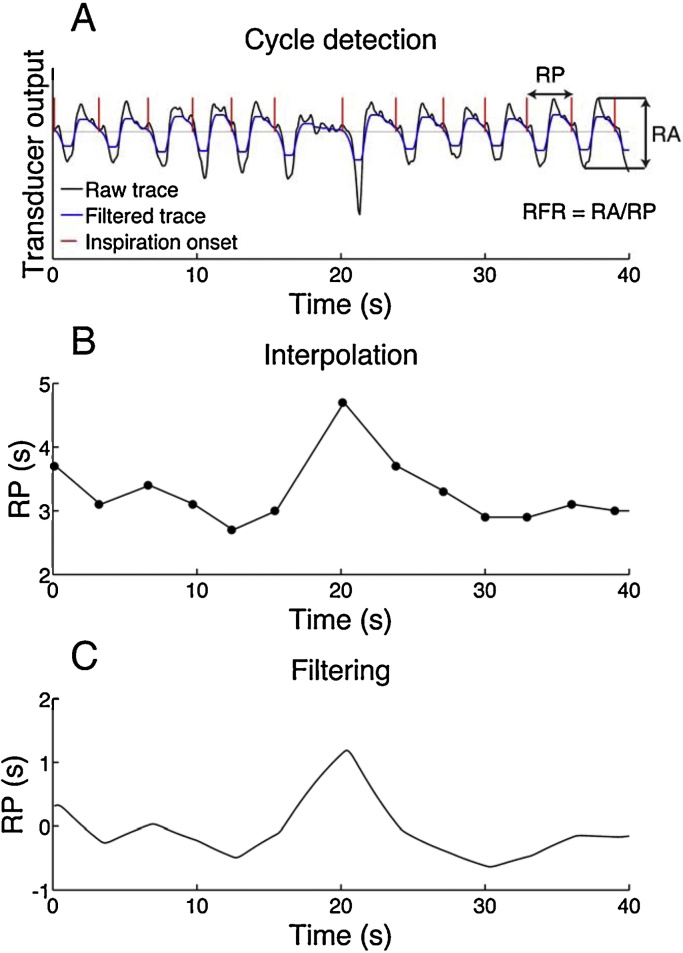
Data preprocessing. Transducer output (A, black) is filtered (blue) to detect sharp pressure changes in the bellows system that mark the onset of inspiration (red). Respiration period (RP) is the cycle duration, respiration amplitude (RA) is the range of the transducer output in a cycle. Both measures, and their quotient respiratory flow rate (RFR), are mapped onto the following inspiration onset (B, black dots), linearly interpolated (B, solid line), and filtered (C). Panels B/C show RP only; RA and RFR are processed analogously. (For interpretation of the references to colour in this figure legend, the reader is referred to the web version of this article.)

In line with previous work (Barry, 1977a), we sought to detect the start of inspiration for temporal assignment of cycle-based measures. For the bellows system, inspiration causes a sharp pressure reduction in the transducer which is well visible to the trained eye. Maximal expiration is visible as a peak in the pressure output which then slowly drifts towards a baseline if the next inspiration does not follow immediately. By trial and error using a selected data set, we developed a simple algorithm to approximate the start of inspiration (Fig. 1A). Specifically, respiration traces were mean-centred, filtered with a bidirectional Butterworth band pass filter and cut-off frequencies of 0.01 Hz and 0.6 Hz, and median filtered over 1 s. A negative zero-crossing was then taken as start of inspiration. After each detected cycle, we imposed a 1 s refractory period, to account for residual signal noise with might cause several zero-crossings on the same cycle. This procedure was compared to visual detection of respiratory cycles by a trained expert (SG) on a random sample of 7 data sets not used for the development of this algorithm. Respiration events were visually marked at the beginning of the inspiration. These time stamps were then compared to the ones found by automatic detection, and each event was categorized as true positive, false positive or false negative. This was done by pairing visually and automatically detected events and searching for missing or redundant pairs. Event pairs with a plausible delay (<1 s) were assumed to be true positive. This procedure revealed 1141 true positives, 5 false positives (i.e. only reported by automated detection) and 6 false negatives (i.e. missed by automated analysis). Hence, sensitivity of the automated method was 99.3%, and the positive predictive value 99.5%. Further, we measured the delay between the visually scored and automatically detected inspiration onset. A median delay of 0.1 s and a standard deviation of 0.1 s was found. Considering the average respiration period (3.4 s in these data sets), the automated method was deemed precise enough.

#### 2.3.3 Analysis with existing methods

For comparison with previous work, we extracted the duration of the respiration cycle into which each event fell (peri-event respiration period, RP), as well as one baseline cycle before (pre-event RP). We either subtracted the baseline (D-RP), or in line with previous work (Barry, 1982) we divided through the baseline (RPQ). The tacit assumption behind the first method is that psychological influences have a linear relation with RP, which is independent from baseline RP. The assumption behind the second method is that this relationship is multiplicative, i.e. it depends on the baseline RP. We also computed RLL. Originally, RLL was defined as the length of the recorded respiration trace over 15 s (Matsuda and Ogawa, 2011) which explains its name “respiration line length”. This approach was later improved by discarding the *x*-component of the respiration trace, and only using the data axis (*y*-component) of the respiration trace for computing RLL (Matsuda and Ogawa, 2011). We used this improved method and projected the respiration trace onto the data axis (*y*-axis) to measure the path length of the ensuing trace over 15 s following each event. Since the current study implemented a between-subjects design (different from the concealed information test where RLL is typically used), we performed a baseline correction by subtracting RLL computed over the 15 s preceding each event, resulting in a difference score D-RLL.

#### 2.3.4 Conversion to continuous data

For each detected respiration cycle we computed three measures: (1) respiration period (RP) is the duration of that cycle. We chose RP rather than the more common respiration rate, its inverse, in analogy to analysis of event-related cardiac responses where heart period has been shown to linearly relate to autonomic nervous system input (Berntson et al., 1995). (2) Respiration amplitude (RA) is the amplitude in rib cage excursion on that cycle, which linearly relates to current tidal volume (VT) (Binks et al., 2007). Since we did not make an attempt to derive the subject-specific proportionality constant relating RA to VT, we use the term RA rather than VT throughout the manuscript. (3) Respiration flow rate (RFR), RA/RP, which is linearly related to tidal volumetric flow rate, i.e. tidal volume per time unit. RFR is computed per cycle with variable duration. Otherwise it is similar to minute volume or RLL which are computed over fixed intervals. Each of these three measures was assigned to the start of the following inspiration cycle and linearly interpolated with 10 Hz sampling frequency. Interpolated data were then filtered twice with a unidirectional first-order Butterworth band pass filter. Initially we used cut-off frequencies of 0.01 Hz and 1 Hz, based on visual inspection of the power spectrum across all data sets and including a frequency band that contained a clearly visible power peak. To determine the optimal filtering method, we repeated the analysis by varying the high-pass frequency (0.001–1 Hz, 0.005–1 Hz, 0.05–1 Hz) and the low-pass frequency (0.01–0.5 Hz, 0.01–2 Hz, 0.01–5 Hz).

#### 2.3.5 Data reduction

Continuous respiration measures were used for general linear models (see below). For derivation of response functions, data were extracted over an interval of [−5 s, 40 s] around each event. We then averaged data across all participants and trials for each experimental condition. This is different from previous work on skin conductance responses and evoked heart period responses, in which we used principal component analysis across all datasets and trials of each experiment condition to summarise responses (Bach et al., 2009, Bach et al., 2010b, Paulus et al., 2016). In the present dataset, however, principal components for all analyses resembled an approximate Fourier series and were therefore not informative about the shape of a respiration response. We therefore chose to use grand averages as for in example in a previous model of fear-conditioned bradycardia (Castegnetti et al., 2016).

#### 2.3.6 General linear modelling

We made the simplifying assumption that cognitive events produce a brief (delta) neural input into a peripheral, linear time-invariant (LTI), system, the output of which is an interpolated respiration time series. In other words, we assumed that the interpolated respiration trace is the convolution of a brief neural input with a canonical response. LTI systems are endowed with two characteristic properties: first, the output does not explicitly depend on time (time invariance), and secondly, the response to several inputs is the sum of the responses to the individual inputs (linearity). Note that in our case, these assumptions relate to the pre-processed data time series rather than to the original respiratory traces. In most real systems, LTI assumptions are only approximately met. In particular the linearity assumption may be brittle since the system will saturate if it receives inputs in quick succession (Paulus et al., 2016); this is why we use long ITI paradigms in this work. This is in contrast to other psychophysiological measures such as skin conductance responses for which the linearity and time invariance assumption directly relate to the biophysical system that generates the data, and can be formally tested in physiological investigations (Bach et al., 2009, Bach et al., 2010b, Bach and Friston, 2013). Finally, also the assumption of a discretised psychological input is a simplification which, although useful, may not truthfully reflect the biophysical reality.

Mathematically, the output *y*(*t*) of a LTI system can be fully described by convolving input *x*(*t*) with the system’s response function *h*(*t*) and can be written as:

$$y\left(t\right)=x\left(t\right)\times h\left(t\right)=\overset{\infty }{\underset{0}{\int }}x\left(t-\tau \right)h\left(\tau \right)\text{d}\tau \text{.}$$

Here, we assume *x*(*t*) as instantaneous (delta) input at event onset and *h*(*t*) as the response function (RF) for respiratory responses. This RF summarises all neural and respiratory processes that finally lead to the respiratory response.

In order to estimate the psychological input into this system, we used a general linear convolution model for each participant and experiment (GLM), in line with previous approaches to analysis of skin conductance responses (Bach et al., 2009), heart period responses (Castegnetti et al., 2016, Paulus et al., 2016), or functional magnetic resonance imaging (Friston et al., 1994). A GLM can be written as

Y=Xβ+ϵ,

where *X* is design matrix in which each column is obtained by convolving impulse functions at event onset with each component of the RF. *Y* is the vector of observations (time series data), *β* is a vector of input amplitude parameters and ϵ is the error that is assumed to be independent and identically distributed. The maximum-likelihood amplitude estimates are computed using the Moore–Penrose pseudoinverse *X^+^*, implemented in the Matlab function pinv:

$$\hat{\beta }=X^{+}Y$$

After model inversion, we tested whether estimates of the input into that convolution system differ from zero, and whether they differentiate experimental conditions. The estimated input can be interpreted as amplitude of a cognitive input, controlling the desired respiration period, tidal volume, or tidal volumetric flow rate.

#### 2.3.7 Model specification

Averaged data from experiments 1–3 was used to construct the RF of the presumed LTI system, by approximating them with a Gaussian function

$$y^{\prime }=Ae^{-{\left(x-\mu \right)}^{2}/2\sigma^{2}}\text{,}$$

with latency parameter *μ*, dispersion parameter *σ*, and amplitude *A*. The best fitting parameters for this Gaussian function were determined by minimizing the residual sum of squares using the Nelder–Mead simplex direct search algorithm implemented in the Matlab function fminsearch (Lagarias et al., 1998). Amplitude parameter *A* was later left free for estimation in the GLM.

In an alternative model, each such RF was complemented with its time derivative analogous to previous models for SCR (Bach et al., 2009, Bach et al., 2013), HPR (Paulus et al., 2016) and functional magnetic resonance imaging (Friston et al., 1994).

**Fig. 2:**
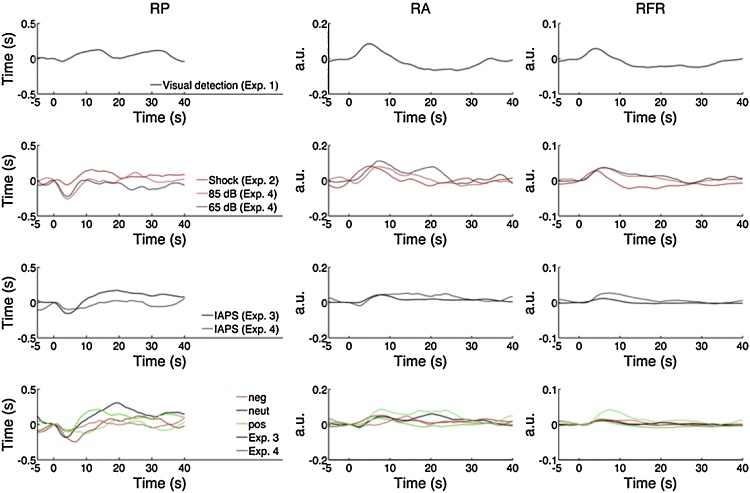
Interpolated responses, averaged across trials and participants, for respiration period (RP), respiration amplitude (RA), and respiratory flow rate (RFR). Responses are grouped by similar manipulations. Row 1: visual detection task (exp. 1). Row 2: aversive stimulation (exps. 2/4) Row 3: picture viewing (exps. 3–4). Row 4: viewing of negative, neutral, and positive IAPS pictures (exps. 3–4). Experiments 1–3 are used to derive canonical response functions, which are validated on independent experiment 4.

**Fig. 3:**
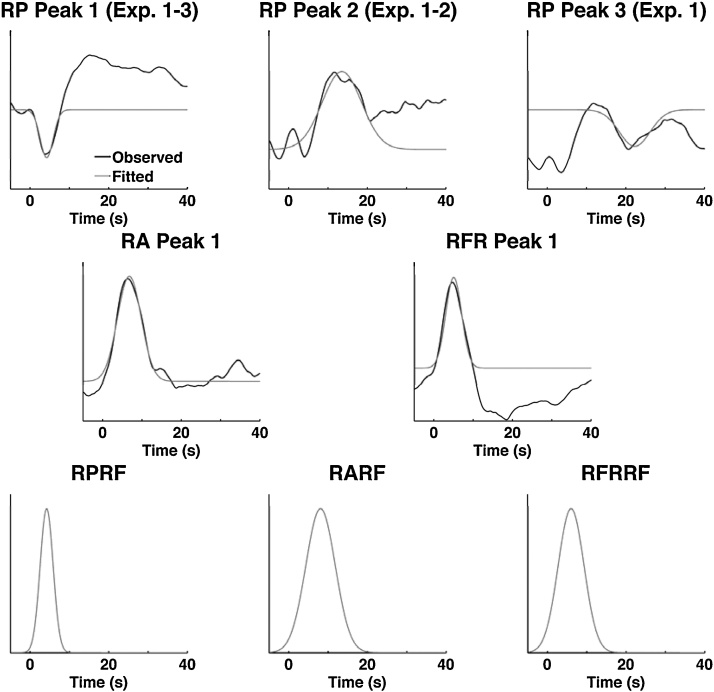
Derivation of canonical response functions (RF) for respiration period (RP), respiration amplitude (RA) and respiratory flow rate (RFR). Response peaks are approximated with Gaussian functions using ordinary least squares minimisation. Lower panels show the final basis functions after filter optimisation.

There appeared to be additional RP response components when considering the 3 experiments individually (Fig. 2). In an exploratory approach, we additionally modelled a later peak in grand average RP data from experiment 2, and an even later peak in combined data from experiments 1–2 (Fig. 3).

## 3 Results

### 3.1 Analysis with existing measures

Table 1 shows descriptive and inference statistics for discrete D-RP/RPQ and D-RLL, for comparison with previous literature. A specific *novelty response* in RP, previously suggested as habituation effect in a visual detection task, was not observed (Table 1, repeated-measures ANOVA within experiment 1). A *defensive response*, previously described as increase in RP of the respiration cycle during which an aversive event occurred, was only observed for RPQ, not D-RP (Table 1, experiment 2). However, this was not specific for defensive responses and also occurred after IAPS pictures (Table 1, experiment 3). Finally, the D-RLL was not specific for *arousal* only, as previously suggested, but instead it increased from baseline in all conditions. None of the discrete measures afforded a differentiation between experimental conditions (ANOVA across experiments, all *p* > 0.10).

### 3.2 Response function

Fig. 2 depicts the mean response in experiments 1–4, for the three different measures. In the first 10 s after an event, RP shows a temporary reduction, while RA and RFR increase. For RP, one can also discern a secondary RP increase around 10 s after an electric shock or visual target but not after pictures, and a tertiary reduction at 20 s after a visual target only. While the overall shape of the response looks somewhat similar between conditions and experiments, there is also variability even between repetitions of the same experimental condition in a different sample (Fig. 2, row 4).

### 3.3 General linear modelling

For the purpose of general linear modelling (GLM), we analytically approximated responses from experiments 1–3 by fitting Gaussian functions to the initial response in RP, RA and RFR, and to the secondary and tertiary responses in RP (Fig. 3). Function parameters for the winning models are summarised in Table 2. Ensuing response functions (RF) were entered into general linear models for each participant, to estimate response amplitudes. For each RF, we evaluated whether it met at least one of three criteria: the presence of an overall effect across experiments 1–3, the ability to discriminate experiments 1–3 in a one-way ANOVA, or the ability to discriminate the three different picture conditions in experiment 3 in a repeated-measures ANOVA. Because RF are evaluated on the data used for their development, this analysis is biased towards finding significant effects. In reverse, if no effect is found in this evaluation for a particular RF, this RF is unlikely to reflect a true response, or to have sensitivity for analysis of independent data sets. RF that met neither criterion were excluded from the validation study.

For RP, the first response function showed an overall negative effect (*t*(70) = −5.42, *p* < 0.0001), reflecting the initial RP decrease shown in Fig. 2. This respiration period response function (RPRF) did not discriminate between the three experiments, or the picture conditions within experiment 3. The other two RFs were added in chronological order, orthogonalised with respect to earlier peaks in the response set. Neither of the two later peaking response functions passed any of the three criteria. The magnitude of the overall effect was not increased by additional modelling of the time derivative of the RPRF or by changing data pre-processing in terms of filter frequencies.

For RA, the response function showed an overall positive effect (*t*(70) = 4.29, *p* < 0.001). Parameter estimates for this respiration amplitude response function (RARF) also showed a significant impact of experimental condition (*F*(2, 68) = 4.36, *p* = 0.017). Modelling the time derivative slightly increased the overall effect and slightly decreased the experiment differences. Modifying filter settings increased both the overall effect and the experimental differences. A band pass filter with cut-off frequencies of 0.001–1 Hz showed highest sensitivity, and this was improved by modelling the time derivative (*t*(70) = 7.10, *p* < 0.001; *F*(2, 68) = 10.72, *p* < 0.001). Exploratory post-hoc tests showed that response to IAPS pictures were smaller than to visual targets (*t*(44) = −5.36, *p* < 0.001) or electric shocks (*t*(43) = −3.50, *p* = 0.001).

Finally, amplitude estimates of the respiratory flow rate response function (RFRRF) showed an overall effect (*t*(70) = 4.64, *p* < 0.001), which was not increased by modelling the time derivative. RFR did not significantly discriminate between any of the experimental conditions. As for RA, the best filter frequency was 0.001–1 Hz, and this was improved by modelling the time derivative (*t*(70) = 7.07, *p* < 0.001). Using the optimal filter and with time derivative, RFR also discriminated between experiments (*F*(2, 68) = 6.80, *p* = 0.003). Exploratory post-hoc tests showed that response to IAPS pictures were smaller than to visual targets (*t*(44) = −4.16, *p* < 0.001) or electric shocks (*t*(43) = −3.07, *p* = 0.004).

### 3.4 Validation

**Fig. 4:**
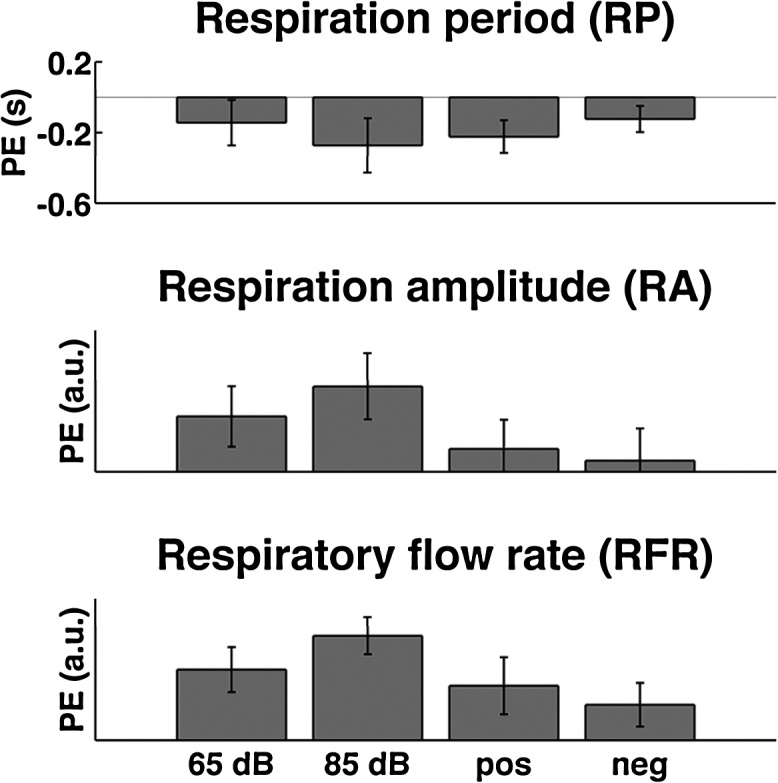
Parameter estimates (PE) for response amplitudes from GLMs in experiment 4 (mean/standard error).

Next, we evaluated the sensitivity of the GLM approach for independent experiment 4 with a within-subjects design. First, we sought to evaluate an overall event-related response across all conditions, as shown in experiments 1–3 for all three measures. Secondly, we sought to replicate the difference between a clearly aversive event (experiment 2) and picture viewing (experiment 3) by contrasting both types of pictures with a loud sound. Third, we note that all events in experiments 1–3 were behaviourally salient in terms of either being aversive (electric shocks), requiring affective evaluation (IAPS pictures) or a specific response (visual detection). We were additionally interested whether the method could be applied to distinguish salient from non-salient events. Thus, we contrasted responses to aversive loud sounds with responses to sounds of low intensity. Results are summarised in Table 3, and mean parameter estimates plotted in Fig. 4. All three measures showed an overall event-related response: a negative RP response, i.e. breathing deceleration, and an increase in RA and RFR. The overall RA response failed to reach significance. Further, RA and RFR were significantly higher for aversive sounds than for picture viewing, as expected from experiments 2–3. None of the measures discriminated between loud and low sounds. We then noted that the mapping between rib cage circumference and lung volume might differ between participants such that our RA and RFR measures contain between-subject variance of no interest, due to individual anatomy (Binks et al., 2007). Previous studies on SCR have suggested an improved sensitivity by *z*-scoring raw data before analysis (Bach, 2014, Bach et al., 2009), which is only possible in a within-subject design. When using *z*-scored data for construction of response functions and model inversion, results were essentially unchanged (Table 2); the overall positive response in RA was significant now.

There is no theoretical reason to expect our analysis to be biased towards detecting an overall event-related effect—parameter estimates are in theory normally distributed around zero under the null hypothesis that no event occurred. To empirically demonstrate this, we entered into our models “non-events”, i.e. we analysed responses at random time points between two events, and contrasted this with the responses to actual events. There was no overall effect for non-events in any of the three measures. All measures showed higher response estimates for events than non-events, although RP failed to reach significance. We note that in experiments 1–3, RA or RFR appeared to return to baseline earlier than RP (Fig. 2), and this may impact on the ability to distinguish events from non-events.

## 4 Discussion

Respiration measures are commonplace in cognitive neuroscience for correction of MR images, but as yet they are rarely used to infer cognitive processes. Here, we present a first attempt at characterising event-related respiratory responses in interpolated data time series, and analysing them in the framework of Psychophysiological Modelling (PsPM). We derive our model from responses to visual detection, aversive stimulation, and viewing of arousing pictures.

As a first result, we observe event-related responses in all three experiments for respiration period (RP), respiration amplitude (RA), and respiratory flow rate (RFR). These responses can be approximated with canonical response functions, and are replicated in a validation experiment. Under the assumption that cognitive processes after an external event elicit a brief neural input into a respiratory LTI system, one can use a general linear modelling approach to estimate the amplitude of that input. Amplitude estimates for RP, RA and RFR are consistently different from zero, and estimates for RA and RFR significantly discriminate between picture viewing and the other two conditions. The overall responses and the impact of experimental condition is confirmed in a validation experiment. As expected, the estimated responses to experimental events differ from those obtained at arbitrary time points, underlining the specificity of the event-related response.

From the evaluated measures, RFR appears particularly useful for two reasons. First, the overall effect and the difference between conditions appear slightly more robust in the independent experiment 4. Secondly, RFR has a clear biological interpretation as it directly relates to gas exchange volume per time unit. It could thus be driven by a central (psychological or neural) input that signals the organism's current oxygen demand. RFR is the quotient of RA and RP. RP not only impacts gas exchange but also has a distinct impact on movement of the organism. This could be particularly important in the context of defensive responses when a prey needs to avoid detection. In such circumstances, RP and RA could be regulated into opposite direction to keep RFR constant, and it may therefore be useful to analyse all three measures. We note that only RP can unambiguously be derived from single chest-belt systems (Binks et al., 2007). It is therefore interesting that the RA and RFR models also generalised to an independent data set. This may indicate that thoracic and abdominal contributions to true RA are relatively constant within any individual and recording session, such that one can usefully approximate true RA with our RA measures up to a linear constant that varies between subjects. However, our proposed model could be improved by using two chest belts for more precise quantification of respiratory volume and volumetric flow rate, or even relating chest-belt measures to accurate spirometric quantities. In the current work, we abstained from doing so as we sought to assess the potential of a simple measure that is available in many cognitive neuroscience laboratories, and standardly used in the context of functional magnetic resonance imaging.

Taken together, our results suggest that interpolated event-related respiratory responses are potentially useful to infer cognitive processes from peripheral measures, and that PsPM is a useful tool for their investigation. How specific this inference may be is an empirical question that we cannot answer in the current paper. It is however encouraging that the main factor influencing respiratory responses appears to be different from factors governing the often used SCR. Specifically, SCR are mainly influenced by stimulus arousal (Boucsein, 2012), but this is not what we find here for RA and RFR. Instead, when comparing experiments 2/3, and conditions within experiment 4, respiratory responses to arousing pictures are significantly smaller than those observed for aversive stimulation (electric shocks or white noise bursts), which is arousing, too. At the same time, visual targets (which are non-arousing) elicit larger responses than arousing pictures when comparing experiments 1/3. On the other hand, responses to the differently valenced negative and positive pictures did not differ in any of the three measures. This pattern suggests that neither arousal nor valence suffices to explain our results. Future studies will aim at elucidating the factors that determine the amplitude of respiratory responses. We note that such knowledge is required to achieve the ultimate goal of inferring cognitive processes from respiratory responses with some specificity.

With the present methodology, one can only estimate the amplitude of a presumed cognitive input into the respiratory system, but it is not possible to determine the type of cognitive process that elicits this input. In situations where the type of cognitive input is suitably constrained, the estimated input amplitude can however be informative. For example, in fear conditioning experiments, one is interested in responses to neutral conditioned stimuli (CS) that only differ in whether or not they predict an aversive outcome. Threat predictions can then be inferred from knowing the estimated amplitude of cognitive input into the SCR or HPR system after a CS (Bach et al., 2010a, Bach et al., 2011, Castegnetti et al., 2016, Staib et al., 2015). Future work will investigate whether respiratory measures can also subserve this goal. For characterising more complex cognitive processes, it has been suggested to use a combination of various psychophysiological measures in a multivariate approach (Stephens et al., 2010).

While we used long ITIs in the present study, this is not normally the case in cognitive neuroscience research. We note that our empirically derived response functions do not extend beyond 25 s after an event (RA) and are as short as 15 s after an event (RP). This means that our method is usable in shorter-ITI paradigms even without using the linearity assumption in the underlying LTI model. To the extent that the linearity assumption is valid, it may be possible to analyse even paradigms with shorter event succession, something that awaits further investigation. Another limitation of the present study is that we use a summary-statistics approach for statistical inference on the group level. That is, we enter single-participant response estimates into a group level *t*-test or ANOVA. This is in keeping with the bulk of psychophysiological literature where peak-scoring estimates are usually averaged within participants before statistical testing. It would in theory be possible to construct a multi-level model that explicitly accounts for within-subject variance. Because subsequent data samples in the interpolated respiration traces are not independent, we note that a multi-level model would require specifying and estimating an auto-regression model, something that could be examined in future work.

We use existing analysis methods to replicate known effects of aversive stimulation on respiration amplitude, and of emotional arousal on RLL, a surrogate for respiratory flow rate. However, none of these measures distinguishes the three experimental manipulations and hence, these previously reported effects do not appear specific in the present investigation. Also, we fail to replicate a presumed respiration period response to novelty. Standard measures rely on shorter post-event time windows than the ones used by our new method. In particular RP is standardly evaluated in one post-event breathing cycle (corresponding to a few seconds) but we show a response that lasts over about 10 s. RLL, a surrogate for RFR, is standardly evaluated over 15 s while we show a response in RFR over 20 s. Note however that we did not aim to directly compare the existing method with our new measures since the true cognitive input is not known in the present study.

To summarise, we demonstrate event-related respiratory responses that are replicable across experiments and distinguish between different experimental conditions. With this work, we hope to inspire renewed interest in the inference of cognitive states from respiratory responses.

## Figures and Tables

**Table 1:** Analysis of respiratory responses with existing measures in experiments 1–3. D-RP: RP difference between pre- and peri-event cycle. RPQ: peri-event RP divided by pre-event RP. D-RLL: RLL difference to baseline. Descriptive data are given as mean (standard error).

Descriptive statistics and *t*-test against zero within each experiment	D-RP (s)	RPQ	D-RLL
	*M* (SEM) *T*	*M* (SEM) *T*	*M* (SEM) *T*
Visual detection (Exp. 1)	0.03 (0.05) *T*(25) = 0.6	4.1 (2.5) *T*(25) = 1.7	1.1 (0.25) *T*(25) = 4.4****
Electric shocks (Exp. 2)	0.08 (0.04) *T*(24) = 2.0	6.2 (2.1) *T*(24) = 3.0**	1.0 (0.28) *T*(24) = 3.6***
IAPS pictures (Exp. 3)	0.21 (0.10) *T*(19) = 2.1*	10.3 (2.5) *T*(19) = 4.1****	0.4 (0.06) *T*(19) = 6.8****

ANOVA across experiments			
Intercept across all groups	*T*(70) = 2.7**	*T*(70) = 4.8****	*T*(70) = 6.3****
Group effect (ANOVA)	*F*(2, 68) = 2.2	*F*(2, 68) = 1.7	*F*(2, 68) = 2.3
Single-trial ANOVA within exp. 1: linear effect of time	*F*(1, 233) = 0.3	*F*(1, 233) = 0.6	*F*(1, 233) = 0.5
Repeated-measures ANOVA within exp. 3: picture type	*F*(2, 38) = 0.3	*F*(2, 38) = 0.0	*F*(2, 38) = 1.8

* *p* < 0.05.

** *p* < 0.01.

*** *p* < 0.005.

**** *p* < 0.001.

**Table 2:** Estimated parameters of the three canonical response functions (RF) for the winning models used for validation, after filter optimisation.

RF	*μ*	*σ*
RPRF	4.20 s	1.65 s
RARF	8.07 s	3.74 s
RFRRF	6.00 s	3.23 s

**Table 3:** Inference statistics on response estimates in experiment 4. We report planned contrasts for estimates from raw respiratory data, and from *z*-scored respiratory data.

	Overall effect (intercept)	Aversive sounds vs. pictures	Aversive vs. non-aversive sounds	Events vs. non-events
Raw data				
RP	*t*(19) = −2.68	*t*(19) = −0.65	*t*(19) = −0.71	*t*(19) = −2.26
*p* = 0.015	n. s.	n. s.	*p* = 0.036
RA	*t*(19) = 1.89	*t*(19) = 2.85	*t*(19) = 0.88	*t*(19) = 2.11
*p* = 0.074	*p* = 0.010	n. s.	*p* = 0.048
RFR	*t*(19) = 4.91	*t*(19) = 2.29	*t*(19) = 1.31	*t*(19) = 3.67
*p* < 0.001	*p* = 0.034	n. s.	*p* = 0.002

*Z*-scored data				
RP	*t*(19) = −3.11	*t*(19) = −0.83	*t*(19) = −0.53	*t*(19) = −2.68
*p* = 0.006	n. s.	n. s.	*p* = 0.015
RA	*t*(19) = 2.74	*t*(19) = 2.78	*t*(19) = 1.18	*t*(19) = 2.62
*p* = 0.013	*p* = 0.012	n. s.	*p* = 0.017
RFR	*t*(19) = 5.17	*t*(19) = 2.35	*t*(19) = 1.35	*t*(19) = 4.05
*p* < 0.001	*p* = 0.030	n. s.	*p* < 0.001

## References

[bib0005] Bach D.R. (2014). A head-to-head comparison of SCRalyze and Ledalab, two model-based methods for skin conductance analysis. Biol. Psychol..

[bib0010] Bach D.R., Friston K.J. (2013). Model-based analysis of skin conductance responses: towards causal models in psychophysiology. Psychophysiology.

[bib0015] Bach D.R., Flandin G., Friston K., Dolan R.J. (2009). Time-series analysis for rapid event-related skin conductance responses. J. Neurosci. Methods.

[bib0020] Bach D.R., Daunizeau J., Friston K.J., Dolan R.J. (2010). Dynamic causal modelling of anticipatory skin conductance responses. Biol. Psychol..

[bib0025] Bach D.R., Flandin G., Friston K.J., Dolan R.J. (2010). Modelling event-related skin conductance responses. Int. J. Psychophysiol..

[bib0030] Bach D.R., Friston K.J., Dolan R.J. (2010). Analytic measures for quantification of arousal from spontaneous skin conductance fluctuations. Int. J. Psychophysiol..

[bib0035] Bach D.R., Weiskopf N., Dolan R.J. (2011). A stable sparse fear memory trace in human amygdala. J. Neurosci..

[bib0040] Bach D.R., Friston K.J., Dolan R.J. (2013). An improved algorithm for model-based analysis of evoked skin conductance responses. Biol. Psychol..

[bib0045] Bach D.R., Seifritz E., Dolan R.J. (2015). Temporally unpredictable sounds exert a context-dependent influence on evaluation of unrelated images. PLoS One.

[bib0050] Barry R.J. (1977). Effect of significance upon indexes of Sokolovs orienting response—new conceptualization to replace OR. Physiol. Psychol..

[bib0055] Barry R.J. (1977). Failure to find evidence of unitary orienting response concept with indifferent low-intensity auditory-stimuli. Physiol. Psychol..

[bib0060] Barry R.J. (1982). Novelty and significance effects in the fractionation of phasic or measures—a synthesis with traditional or theory. Psychophysiology.

[bib0065] Berntson G.G., Cacioppo J.T., Quigley K.S. (1995). The metrics of cardiac chronotropism: biometric perspectives. Psychophysiology.

[bib0070] Berntson G.G., Quigley K.S., Lozano D., Cacioppo J.T.T., Tassinary L.G., Berntson G.G. (2007). Cardiovascular psychophysiology. Handbook of Psychophysiology.

[bib0075] Binks A.P., Banzett R.B., Duvivier C. (2007). An inexpensive, MRI compatible device to measure tidal volume from chest-wall circumference. Physiol. Meas..

[bib0080] Boiten F.A., Frijda N.H., Wientjes C.J. (1994). Emotions and respiratory patterns: review and critical analysis. Int. J. Psychophysiol..

[bib0085] Boucsein W. (2012). Electrodermal Activity.

[bib0090] Bradley M.M., Codispoti M., Cuthbert B.N., Lang P.J. (2001). Emotion and motivation I: defensive and appetitive reactions in picture processing. Emotion.

[bib0095] Castegnetti G., Tzovara A., Staib M., Paulus P.C., Hofer N., Bach D.R. (2016). Modeling fear-conditioned bradycardia in humans. Psychophysiology.

[bib0100] Friston K.J., Jezzard P., Turner R. (1994). Analysis of functional MRI time-series. Hum. Brain Mapp..

[bib0105] Friston K.J., Ashburner J.T., Kiebel S.J., Nichols T.E., Penny W.D. (2008). Statistical Parametric Mapping: The Analysis of Functional Brain Images.

[bib0110] Glover G.H., Li T.Q., Ress D. (2000). Image-based method for retrospective correction of physiological motion effects in fMRI: RETROICOR. Magn. Reson. Med..

[bib0115] Grassi M., Caldirola D., Di Chiaro N.V., Riva A., Dacco S., Pompili M., Perna G. (2014). Are respiratory abnormalities specific for panic disorder? A meta-analysis. Neuropsychobiology.

[bib0120] Grassmann M., Vlemincx E., von Leupoldt A., Van den Bergh O. (2015). The role of respiratory measures to assess mental load in pilot selection. Ergonomics.

[bib0125] Hutton C., Josephs O., Stadler J., Featherstone E., Reid A., Speck O., Bernarding J., Weiskopf N. (2011). The impact of physiological noise correction on fMRI at 7 T. NeuroImage.

[bib0130] Lagarias J.C., Reeds J.A., Wright M.H., Wright P.E. (1998). Convergence properties of the Nelder–Mead simplex method in low dimensions. SIAM J. Optim..

[bib0135] Lang P.J., Bradley M.M., Cuthbert B.N. (2005). International affective picture system (IAPS): Affective ratings of pictures and instruction manual. Technical Report A-6.

[bib0140] Lorig T.S., Cacioppo J.T.T., Tassinary L.G., Berntson G.G. (2007). The respiratory system. Hanndbook of Psychophysiology.

[bib0145] Matsuda I., Ogawa T. (2011). Improved method for calculating the respiratory line length in the Concealed Information Test. Int. J. Psychophysiol..

[bib0150] Pappens M., Schroijen M., Van den Bergh O., Van Diest I. (2015). Retention of perceptual generalization of fear extinction. Int. J. Psychophysiol..

[bib0155] Paulus P.C., Castegnetti G., Bach D.R. (2016). Modelling event-related heart period response. Psychophysiology.

[bib0160] Ritz T., Kullowatz A., Goldman M.D., Smith H.J., Kanniess F., Dahme B., Magnussen H. (2010). Airway response to emotional stimuli in asthma: the role of the cholinergic pathway. J. Appl. Physiol. (1985).

[bib0165] Ritz T., Bobb C., Griffiths C. (2014). Predicting asthma control: the role of psychological triggers. Allergy Asthma Proc..

[bib0170] Staib M., Castegnetti G., Bach D.R. (2015). Optimising a model-based approach to inferring fear learning from skin conductance responses. J. Neurosci. Methods.

[bib0175] Stephens C.L., Christie I.C., Friedman B.H. (2010). Autonomic specificity of basic emotions: evidence from pattern classification and cluster analysis. Biol. Psychol..

[bib0180] Van Diest I., Janssens T., Bogaerts K., Fannes S., Davenport P.W., Van Den Bergh O. (2009). Affective modulation of inspiratory motor drive. Psychophysiology.

[bib0185] Vlemincx E., Van Diest I., Van den Bergh O. (2014). Emotion, sighing, and respiratory variability. Psychophysiology.

[bib0190] Wientjes C.J., Grossman P. (1998). Respiratory psychophysiology as a discipline: introduction to the special issue. Biol. Psychol..

[bib0195] Wuyts R., Vlemincx E., Bogaerts K., Van Diest I., Van den Bergh O. (2011). Sigh rate and respiratory variability during normal breathing and the role of negative affectivity. Int. J. Psychophysiol..

